# Involvement of potential pathways in malignant transformation from Oral Leukoplakia to Oral Squamous Cell Carcinoma revealed by proteomic analysis

**DOI:** 10.1186/1471-2164-10-383

**Published:** 2009-08-19

**Authors:** Zhi Wang, Xiaodong Feng, Xinyu Liu, Lu Jiang, Xin Zeng, Ning Ji, Jing Li, Longjiang Li, Qianming Chen

**Affiliations:** 1 State Key Laboratory of Oral Diseases, West China Hospital of Stomatology, Sichuan University, Chengdu, 610041, Sichuan, PR China; 2 The State Key Laboratory of Biotherapy, West China Hospital, Sichuan University, Chengdu, 610041, PR China

## Abstract

**Background:**

Oral squamous cell carcinoma (OSCC) is one of the most common forms of cancer associated with the presence of precancerous oral leukoplakia. Given the poor prognosis associated with oral leukoplakia, and the difficulties in distinguishing it from cancer lesions, there is an urgent need to elucidate the molecular determinants and critical signal pathways underlying the malignant transformation of precancerous to cancerous tissue, and thus to identify novel diagnostic and therapeutic target.

**Results:**

We have utilized two dimensional electrophoresis (2-DE) followed by ESI-Q-TOF-LC-MS/MS to identify proteins differentially expressed in six pairs of oral leukoplakia tissues with dysplasia and oral squamous cancer tissues, each pair was collected from a single patient. Approximately 85 differentially and constantly expressed proteins (> two-fold change, P < 0.05) were identified, including 52 up-regulated and 33 down-regulated. Gene ontological methods were employed to identify the biological processes that were over-represented in this carcinogenic stage. Biological networks were also constructed to reveal the potential links between those protein candidates. Among them, three homologs of proteosome activator PA28 a, b and g were shown to have up-regulated mRNA levels in OSCC cells relative to oral keratinocytes.

**Conclusion:**

Varying levels of differentially expressed proteins were possibly involved in the malignant transformation of oral leukoplakia. Their expression levels, bioprocess, and interaction networks were analyzed using a bioinformatics approach. This study shows that the three homologs of PA28 may play an important role in malignant transformation and is an example of a systematic biology study, in which functional proteomics were constructed to help to elucidate mechanistic aspects and potential involvement of proteins. Our results provide new insights into the pathogenesis of oral cancer. These differentially expressed proteins may have utility as useful candidate markers of OSCC.

## Backgound

Oral, head, and neck squamous cellular carcinoma is one of the most common forms of cancer associated with the presence of precancerous lesions. It is now believed that OSCC follows a similar pattern in its development, and thus is preceded by precancerous lesions, among which oral leukoplakia (OLK) is the most common type. The World Health Organization (WHO) first defined oral leukoplakia as a white plaque that could not be characterized clinically or pathologically as any other disease in oral mucosa. The malignant potential of oral leukoplakia was evidenced by the progression from metaplasia without dysplasia to low grade dysplasia, high grade dysplasia, and ultimately to invasive carcinoma [1]. The risk of developing malignancies is 8–10 times higher in people who have oral leukoplakia than people who do not [2]. The risk is also increasing with the series of dysplasia stages [3]. There is an urgent need to elucidate the molecular determinants and key signal pathways underlying the malignant transformation from precancerous to cancerous tissue, and thus to identify novel diagnostic and therapeutic targets.

Proteomics is an established molecular profiling technology that may significantly accelerate human cancer research. Recently, a lot of progress has been made in oral cancer proteomics generating some potential applications in this emerging field. This technology platform has been utilized to discover highly sensitive and specific protein markers for oral cancer diagnosis and prognosis by comparing the protein profiles of cancer cells [4,5], tissues [6], plasma [7], and saliva[8,9] with appropriate controls. However, there are fewer reports about discrimination of protein expression profiles between tumor and precancerous lesion with different stages of dysplasia.

The development of bioinformatics tools has allowed the compilation of searchable genomic and proteomic databases accessible via the Internet. Among them, the application of Gene Ontology (GO) and the pathway analysis was considered as a powerful tool in systematic biology for elucidating the complexity of expression profiles in cellular processes. The term of GO describes the role of a given gene in a biological process, its molecular function and cellular component. Each gene is provided with different levels of GO terms, ranging from high-level, broadly descriptive terms to very low-level, highly specific terms [10]. Thus, profiling the expression data based on GO will provide another dimension for understanding the key regulatory processes in oral cancer. The application of the pathway analysis reveals the interactions between the proteins, thus quickly generating new insights into potential complex molecular mechanisms underlying disease related processes [11].

In this study, we have evaluated protein expression differences to identify potential biomarkers of disease progress from oral leukoplakia to OSCC in order to gain further insight into potential mechanisms underlying these transformations. Six pairs of protein lysates were obtained from six patients. The tissues were analyzed by two-dimensional gel electrophoresis, followed by ESI-Q-TOF tandem mass spectrometry. GO analysis was applied to identify biological processes over-represented in the carcinogenesis. Biological networks were also constructed to reveal the potential links between the protein candidates. By using this approach, new therapeutic targets or protein markers can possibly be identified to improve patient survival.

## Results

### 2-DE profiling of OSCC and the oral leukoplakia tissues

**Figure 1:**
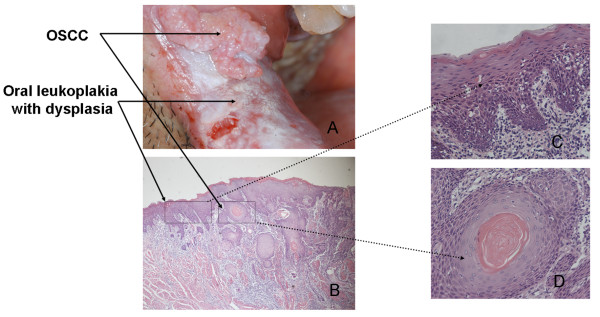
**Representative clinical and histological pictures**. Representative clinical (A) and histological pictures (B, C, D) showing a pair of OSCC and oral leukoplakia tissues with dysplasia obtained from the same patient.

**Figure 2:**
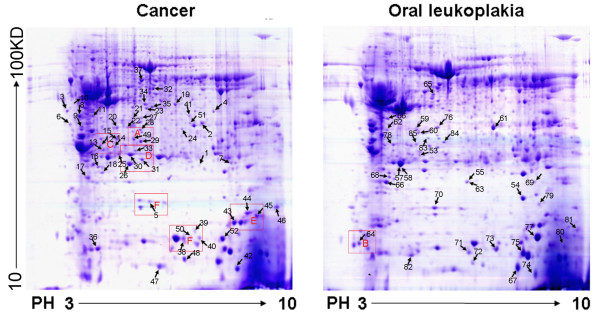
**Representative two-dimensional maps marked altered proteins**. Representative two-dimensional maps of a pair of OSCC cancer tissue and precancerous oral leukoplakia tissue from the same patient. The proteins were separated on a pH 3–10 nonlinear IPG strip, followed by a 12% SDS-polyacrylamide gel, as stated under Methods. The gel was Coomassie-blue stained and the spots were analyzed by ESI-Q-TOF-LC-MS/MS. Arrows indicate identified protein spots significantly and consistently altered between carcinoma tissue and control tissue. 11 boxed proteins were selected as examples showing the consistent expression changes in enlarged form in additional file 1.

A total of 6 pairs of OSCC tumor tissues and the oral leukoplakia control tissues were obtained from 6 patients. Figure 1 showed their representative clinical photos and HE-staining histographs. 2-DE with immobilized pH gradients was performed to study the expression patterns of proteins extracted from both tissues, and each sample was analyzed twice to ensure the reproducibility. Figure 2 showed representative 2-DE patterns obtained from the paired tissues. After automatic spot detection, background subtraction, and volume normalization, 859 ± 68 protein spots in OSCC tissue, and 844 ± 56 protein spots in control tissues were detected. Of these spots, 730 (85%) in OSCC tissues and 708 (84%) in control tissues were reproducibly detected in all of the twelve runs, and only the reproducibly detected spots were subjected to statistical analysis(p < 0.05). As a result, 85 protein spots showed more than two fold changes in at least 4 of the twelve repeats (marked in figure 2) while 68 proteins (80%) repeated in more than 8 of 12 pairs.11 proteins were selected as examples (boxed in figure 2) showing the consistent expression changes in enlarged form (see additional file 1).

### Identification of putative OSCC biomarkers

**Figure 3:**
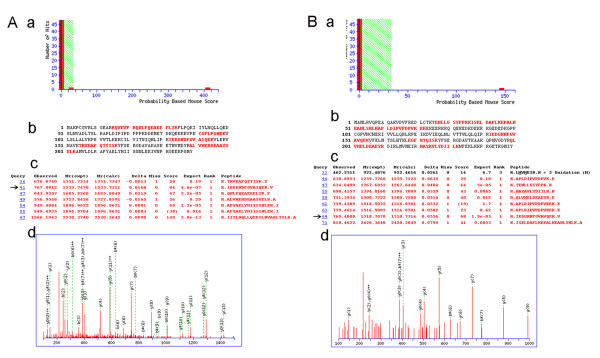
**Results of PA28 b and a as the representative protein identified using ESI-Q-TOF-MS/MS**. A a b, output of the database searching by the MASCOT program using MS/MS data used in the identification of PA28 b. The matched peptides were shown in bold red. A c d, MS/MS spectrum of parent ions with m/z values of 1533.7311(arrow marked). B a b, output of the database searching by the MASCOT program using MS/MS data used in the identification of PA28 a. B c d, MS/MS spectrum of parent ions with m/z values of 1518.7314(arrow marked).

As shown in Table 1, a total of 85 differentially expressed proteins, including 52 up-regulated and 33 down-regulated proteins, were identified. Proteasome activator complex PA28 a and b were chosen for further validation. They both exhibited a high expression level in cancer tissues (4–6 fold increase) when compared with the precancerous OLK tissues. The mass spectra for both were shown in Figure 3.

**Table 1:** Summary of protein alterations

Spot number^a^	Swissprot No.^b^	Protein name	*M*r^c^	PI^d^	Score^e^	Peptides^f^	Mean fold^g^	Repeat^h^
1	*Q5T2P8	Annexin A8-like protein 1	37086	5.45	221	13	-3.09 ± 0.24	8

2	P13928	Annexin A8	36842	5.56	269	11	-2.98 ± 0.26	8

3	Q01105	Protein SET	33469	4.23	107	5	5.89 ± 0.52	6

5	Q99497	Protein DJ-1	20,050	6.33	187	5	3.22 ± 0.43	8

4	P52907	F-actin-capping protein subunit alpha-1	32902	5.45	118	6	2.23 ± 0.31	

6	Q13765	Nascent polypeptide-associated complex subunit alpha	23370	4.52	451	10	4.37 ± 0.51	8

7	*P24534	Elongation factor 1-beta	24748	4.5	45	1	2.42 ± 0.3	8

8	*P02533	Keratin, type I cytoskeletal 14	51589	5.09	432	17	4.32 ± 0.56	8

9	P09493	Tropomyosin alpha-1 chain	32746	4.69	162	15	6.27 ± 0.38	8

10	P08107	Heat shock 70 kDa protein 1	70009	5.48	234	8	2.33 ± 0.28	8

11	*P07910	Heterogeneous nuclear ribonucleoproteins C1/C2	33650	4.95	140	9	2.95 ± 0.35	6

12	P10809	60 kDa heat shock protein, mitochondrial precursor	61016	5.7	103	7	-4.32 ± 0.58	10

13	P12004	Proliferating cell nuclear antigen	28750	4.57	113	4	7.95 ± 0.38	12

14	P40261	Nicotinamide N-methyltransferase	30011	5.56	59	10	-4.06 ± 0.58	8

15	Q9UL46	PA28 b	27515	5.44	759	28	5.88 ± 0.68	12

16	P52566	Rho GDP-dissociation inhibitor 2	23031	5.1	248	11	3.14 ± 0.28	12

17	* P02679	Fibrinogen gamma chain precursor	51479	5.37	326	18	-3.05 ± 0.42	8

18	P52565	Rho GDP-dissociation inhibitor 1	23193	5.02	211	9	3.44 ± 0.32	12

19	* P05112	Interleukin-4 precursor	17481	9.17	81	1	4.12 ± 0.62	4

20	*P68363	Tubulin alpha-1B chain	50120	4.94	325	16	3.86 ± 0.31	10

21	P05388	60S acidic ribosomal protein P0	34252	5.71	68	6	5.24 ± 0.71	6

22	Q07021	Complement component 1 Q	31742	4.74	309	6	2.12 ± 0.49	8

23	*P02675	Fibrinogen beta chain precursor	56577	8.54	236	18	13.65 ± 1.44	8

24	*P01009	Alpha-1-antitrypsin precursor	46707	5.37	99	2	4.22 ± 0.65	4

25	P09936	Ubiquitin carboxyl-terminal hydrolase isozyme L1	25151	5.33	326	22	11.5 ± 0.84	4

26	P09211	Glutathione S-transferase P	23341	5.43	468	16	5.44 ± 0.24	12

27	P07437	Tubulin beta chain	50095	4.78	197	12	5.67 ± 1.02	10

28	P14618	Pyruvate kinase isozymes M1/M2	58470	7.96	285	18	5.22 ± 0.29	12

29	Q06323	PA28 a	28705	5.78	147	9	4.44 ± 0.25	12

30	P04792	heat shock 27 kDa protein 1	22768	5.98	565	42	5.56 ± 0.37	10

32	P30048	peroxiredoxin 3	27675	7.67	367	28	3.04 ± 0.4	10

31	*P11142	Heat shock cognate 71 kDa protein	70854	5.37	134	7	5.26 ± 0.79	8

33	Q13162	Peroxiredoxin-4	30521	5.86	95	8	4.74 ± 0.22	12

34	P36952	Serpin B5 precursor	42111	5.72	324	19	5.26 ± 0.47	12

35	Q15019	Septin-2	41461	6.15	191	6	3.88 ± 0.65	8

36	Q01469	Fatty acid-binding protein, epidermal	15155	6.6	41	3	6.24 ± 0.25	12

37	*P19013	Keratin, type II cytoskeletal 4	57649	6.25	1023	28	3.12 ± 0.70	4

38	P60709	Actin, cytoplasmic 1	42052	5.29	806	28	5.88 ± 1.02	6

39	Q969H8	Uncharacterized protein C19orf10	18897	6.2	167	11	3.23 ± 0.56	8

40	P47929	Galectin-7	15066	7.03	730	22	6.44 ± 0.26	12

41	* P06733	Alpha-enolase	47139	7.01	1000	27	4.33 ± 0.49	8

42	P60903	Protein S100-A10	11196	6.82	149	18	5.45 ± 0.62	6

43	P62937	Peptidyl-prolyl cis-trans isomerase A	18229	7.68	2056	75	5.68 ± 0.85	6

44	P23528	Cofilin-1	18491	8.22	1677	35	3.11 ± 0.42	8

45	*P22392	Nucleoside diphosphate kinase B	17287	8.52	235	20	4.44 ± 0.23	12

46	Q01995	Transgelin	22596	8.87	238	16	3.28 ± 0.19	12

47	P01834	Ig kappa chain C region	11602	5.58	228	24	3.44 ± 0.65	10

48	P31949	Protein S100-A11	10173	6.56	185	8	5.56 ± 0.75	8

49	P62736	Actin, aortic smooth muscle	41982	5.23	119	6	3.67 ± 0.55	8

50	Q64133	Amine oxidase [flavin-containing] A	60157	7.94	111	1	7.56 ± 0.8	6

51	P47755	F-actin-capping protein subunit alpha-2	32929	5.57	119	11	3.24 ± 0.45	8

52	Q96A32	Myosin regulatory light chain 2, skeletal muscle isoform	19116	4.91	517	33	5.73 ± 0.35	12

53	*P02743	Serum amyloid P-component	25485	6.1	140	5	-2.34 ± 0.45	6

54	P02511	Alpha-crystallin B chain	20146	6.76	874	34	-9.5 ± 1.05	10

55	P61088	Ubiquitin-conjugating enzyme E2 N	17184	6.1	3	86	-3.23 ± 0.45	10

56	P08758	Annexin A5	35914	4.94	496	10	-5.74 ± 0.28	12

57	P62942	FK506-binding protein 1A	11943	7.88	51	2	-2.56 ± 0.45	12

58	P68871	Hemoglobin subunit beta	16102	6.75	996	45	-2.02 ± 0.52	8

59	Q14152	Eukaryotic translation initiation factor 3 subunit I	36479	5.38	190	15	-2.96 ± 0.47	10

60	P06702	Protein S100-A9	13234	5.71	548	23	-6.42 ± 0.58	12

61	P04083	Annexin A1	38690	6.57	2528	60	-5.03 ± 0.28	12

62	P55083	Microfibril-associated glycoprotein 4	28972	5.38	95	1	-3.69 ± 0.55	8

63	P40925	Malate dehydrogenase cytoplasmic	36,403	6.91	4	86	-2.87 ± 0.62	8

64	P27482	Calmodulin-like protein 3	16937	4.3	1562	55	-4.22 ± 0.35	10

65	* P61158	Actin-related protein 3	47341	5.61	93	9	-2.14 ± 0.45	8

66	*P02768	Serum albumin precursor	71317	5.92	856	52	-7.67 ± 0.85	10

67	P05109	Protein S100-A8	10828	6.51	136	13	-3.34 ± 0.55	12

68	*P05976	Myosin light chain 1, skeletal muscle isoform	21132	4.97	881	49	-13.5 ± 0.85	12

69	P30086	Phosphatidylethanolamine-binding protein 1	21044	7.01	210	5	-3.78 ± 0.65	6

70	P11177	Pyruvate dehydrogenase E1 component subunit beta,	39208	6.2	64	5	-5.35 ± 0.25	12

71	P31151	Protein S100-A7	11564	6.28	131	7	-6.23 ± 0.44	12

72	Q96FQ6	Protein S100-A16	11794	6.28	68	4	-7.14 ± 0.86	8

73	P49773	Histidine triad nucleotide-binding protein 1	13793	6.43	40	3	-3.23 ± 0.52	8

74	P69905	Hemoglobin subunit alpha	15305	8.72	769	43	-7.68 ± 1.04	8

75	P04080	Cystatin-B	11133	6.96	378	28	-3.89 ± 0.18	12

76	P07195	L-lactate dehydrogenase B chain	36615	5.71	246	12	-2.69 ± 0.55	6

77	P02144	Myoglobin	17173	7.14	2284	53	-19.22 ± 1.38	12

78	O00299	Chloride intracellular channel protein	26906	5.09	405	16	-2.23 ± 0.48	6

79	P07737	Profilin-1	15216	8.44	1252	53	-5.69 ± 0.35	12

80	O95994	Anterior gradient protein 2 homolog	19967	9.03	292	10	-2.87 ± 0.52	8

81	*P62988	Ubiquitin	18560	6.56	112	5	-5.22 ± 0.43	6

82	P45378	Troponin T, fast skeletal muscle	31805	5.71	233	10	-2.99 ± 0.45	8

83	P12429	Annexin A3	36353	5.63	169	10	-4.41 ± 0.28	12

84	P09525	Annexin A4	35860	5.84	694	20	-2.88 ± 0.56	8

85	P35232	Prohibitin	29843	5.57	214	14	-3.24 ± 0.45	8

*a *The spot number correspond to those on 2-DE images shown in Fig. 2

*b *Swiss-Prot accession number.

*c *Theoretical molecular mass (kDa) and pI from the ExPASy database.

*d *Theoretical pI from the ExPASy database.*e *The number of unique peptides identified by MS/MS sequencing.

*f *Probability-based MOWSE (molecular weight search) scores. g Expression change level in OSCC tumor tissue compared with control (+, increase in tumor;-, decrease in tumor). Data were represented as mean ± SD

*h *frequency of the up-regulation (or down-regulation) of the 85 proteins identified in the six sample pairs.

* new proteins obtained by analysis of expression pattern through web-based tool (GOTree)

### Finding functional enrichment in transformation from oral leukoplakia to OSCC through gene ontology

**Figure 4:**
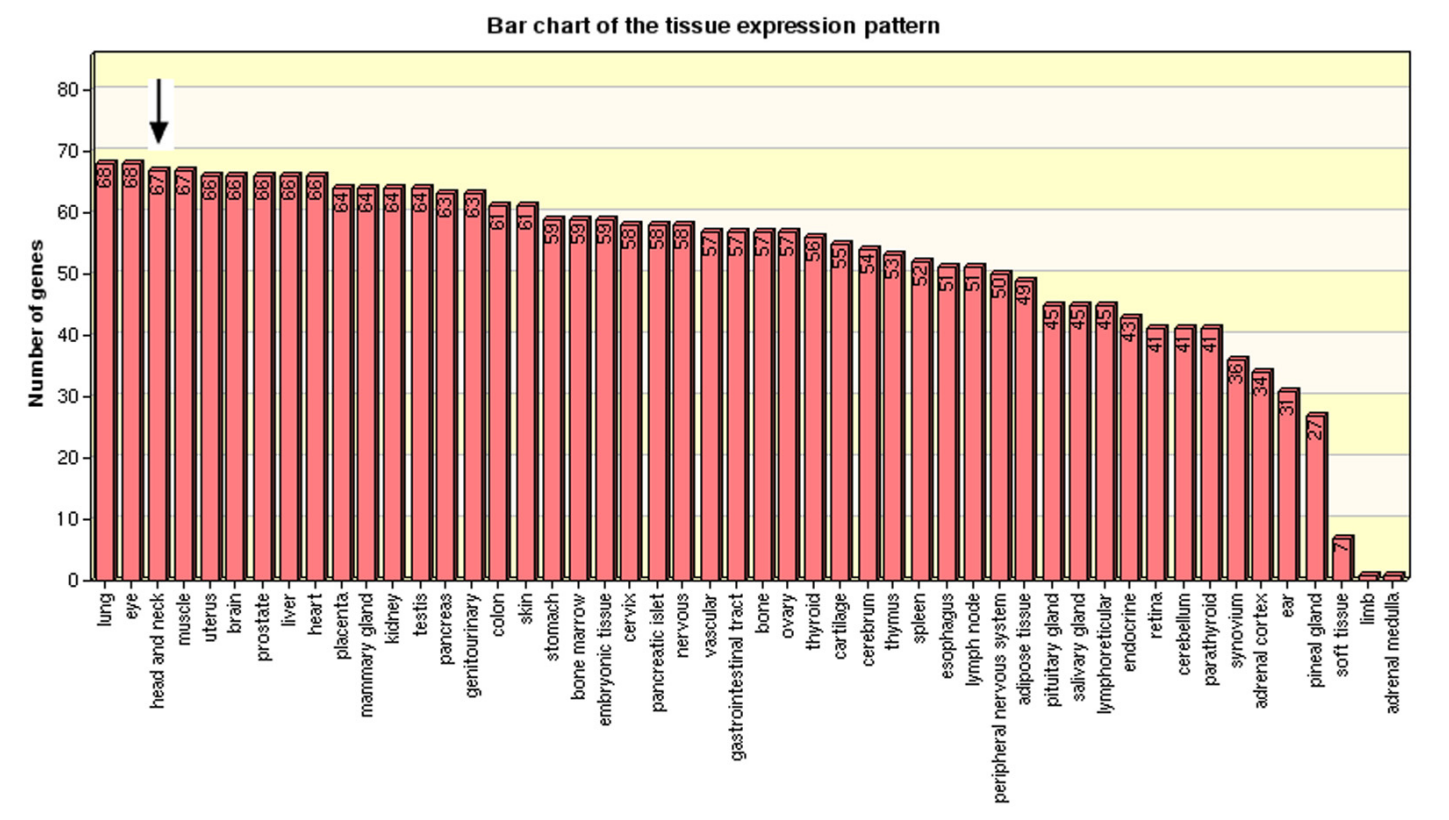
**Expression Profiling Diagram**. The diagram was constructed with the use of the Ingenuity Pathway Analysis software as described in Materials and Methods and in Results. 18 novel proteins were shown to be firstly expressed in head and neck.

**Figure 5:**
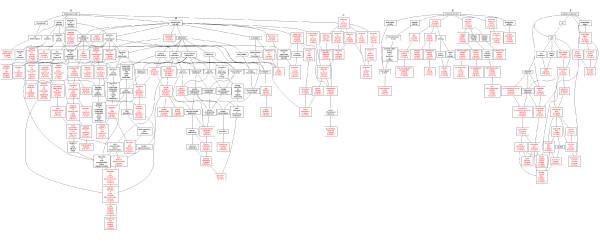
**Directed acyclic graph (DAG) view of the enriched GO categories in the transformation process from precancerous oral leukoplakia to OSCC**. The enriched GO categories were brought together and visualized as a DAG. Categories in red were enriched, while those in black were non-enriched parents . A) biological process, B) physiological process C) response to stimulus D) molecular function E) cellular component.

Ontological methods were employed to structure the biological processes that were over-represented in the carcinogenic stage from oral leukoplakia to infiltrative oral cancer. A web-based tool (GOTree) was employed in which the biological process of the proteins encoded by the genes is scored. Those with a level higher than 4 were highlighted. The observed versus the expected number of genes in the GO categories using a *Homo sapiens *reference data set was analyzed. 18 novel proteins, each marked with an asterisk in Table 1, were demonstrated to be expressed in head and neck by the analysis of tissue expression profile using GOTree, and their bar chart is shown in Figure 4. X-axis represented the different tissue. The Y-axis means the proteins has been reported in corresponding tissue. Figure 5 shows the tree-like structure with their respective molecular functions, cellular components and biological processes. Taken the biological processes as an example, it involved six processes, the processes responsive to stimulus (including heat shock 70 kDa, 60 kDa and 27 kDa proteins, tubulin, heat shock 70 kDa protein 8, annexin A5, proliferating cell nuclear antigen, S100A7,8,9, annexin A1 and A8, and interleukin 4); physiological response(including serpin peptidase inhibitor, Rho GDP dissociation inhibitor beta, fibrinogen, PA28 a and b); negative regulation of biological process (including capping protein muscle, annexin A4, SET translocation, glutathione S-transferase, S100A11, annexin A1, non-metastatic cells 2 protein, and prohibitin); locomotion proteins (including annexin A1, SERPINB5, Rho GDP dissociation inhibitor (GDI) alpha, tropomyosin 1, and heat shock 27 kDa protein 1); cell death process (including heat shock 27 and 70 kDa protein, annexin A4 annexin A5, galectin 7, and prohibitin); and coagulation (including annexin A4, A5, A8, and fibrinogen).

### Link of the Proteins to Biological Pathways

**Figure 6:**
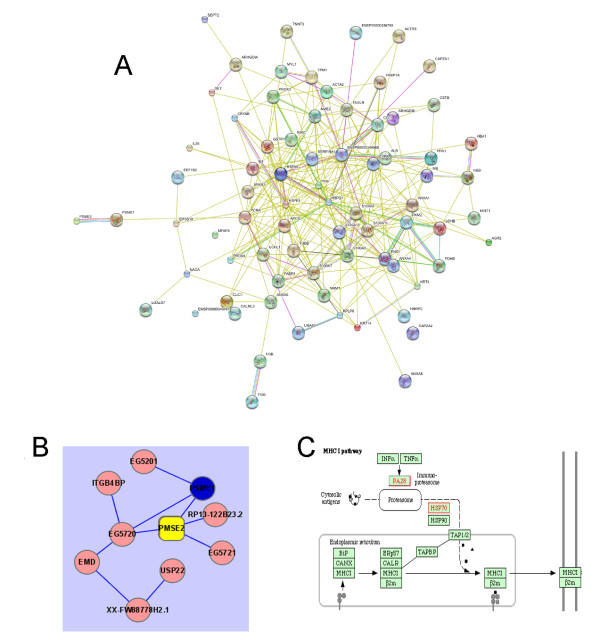
**Organization of dysregulated protein genes into common pathways in the transformation process from precancerous oral leukoplakia to OSCC by Pathway Studio**. Proteins with their SWISS-PROT number were loaded into Pathway Studio software , and analyzed using the Pathway Studio program with the ResNet 2.5 database. (A) 85 protein genes with a high confidence index of interactions were included in the pathway layout graph. (B) The indirect interaction of eight proteins with PA28 a and b (gene name: PSME1 and 2) as reported in the cancer-related literature. (C) The key proteins in MHC antigen presenting pathway, among which PA28, proteosome, HSP70 and HSP90 have been detected up-regulated in our study. Each node represents either a protein entity or a control mechanism of the interaction. Connecting lines between the protein symbols indicate interactions; different types of interactions are denoted by symbols on the lines. Green square indicates regulation; purple square, binding; blue square, expression; orange circle, protein modification; red diamond, metabolism; green circle, promoter binding; yellow triangle, transport; "+" in gray circle, positive effect; and "-" in gray circle, negative effect.

To establish an overview of the interactions among differentially regulated proteins, and to prioritize proteins and pathways for further evaluation, we used the Pathway Studio software to explore the associations between differentially regulated proteins, essentially based on the available knowledge about eukaryotic molecular interactions documented in the ResNet database. Notably, central nodes of proteins of the biological network, generated by the Pathway Architect assembly of GO designations for the most prominently over-represented genes, were shared in oral leukoplakia tissues and tumor tissues including S100 family (A7, 8, 9, 10, and 11), HSP family (HSPB1 and HSPA8), ANX family (A1, 3, 4, and 5), tumor metastasis suppressor NME2 and Rho GDP dissociation inhibitor alpha and beta (ARHGDIA and B), pyruvate kinase (PKM2), transgelin (TAGLN), glutathione S-transferase (GSTP1), SERPINA1, PCNA, and so on (Figure 6A). Those proteins and their interactive pathways merit further study for their roles in oral carcinogenesis.

### Biological Pathways related to PA28

Pathway analysis was also used to reveal the protein interactions and potential pathways of PA28. The result showed that some proteins interacted with PA28 either directly or indirectly, which was not identified by the proteomics approach due to their low abundance (Figures 6B). It was generally accepted that PA28ab contributes to Class I presentation in immune tissues. Our results also reinforced the connection between PA28ab and cellular immunity as the proteins involved in the MHC-I antigen presenting pathway like PA28, proteosome, HSP70 and HSP90 have been detected to be up-regulated (Table 1, Figures 6C).

### Overexpression of PA28 in OSCC Cancer Cells and Cancer Tissues

**Figure 7:**
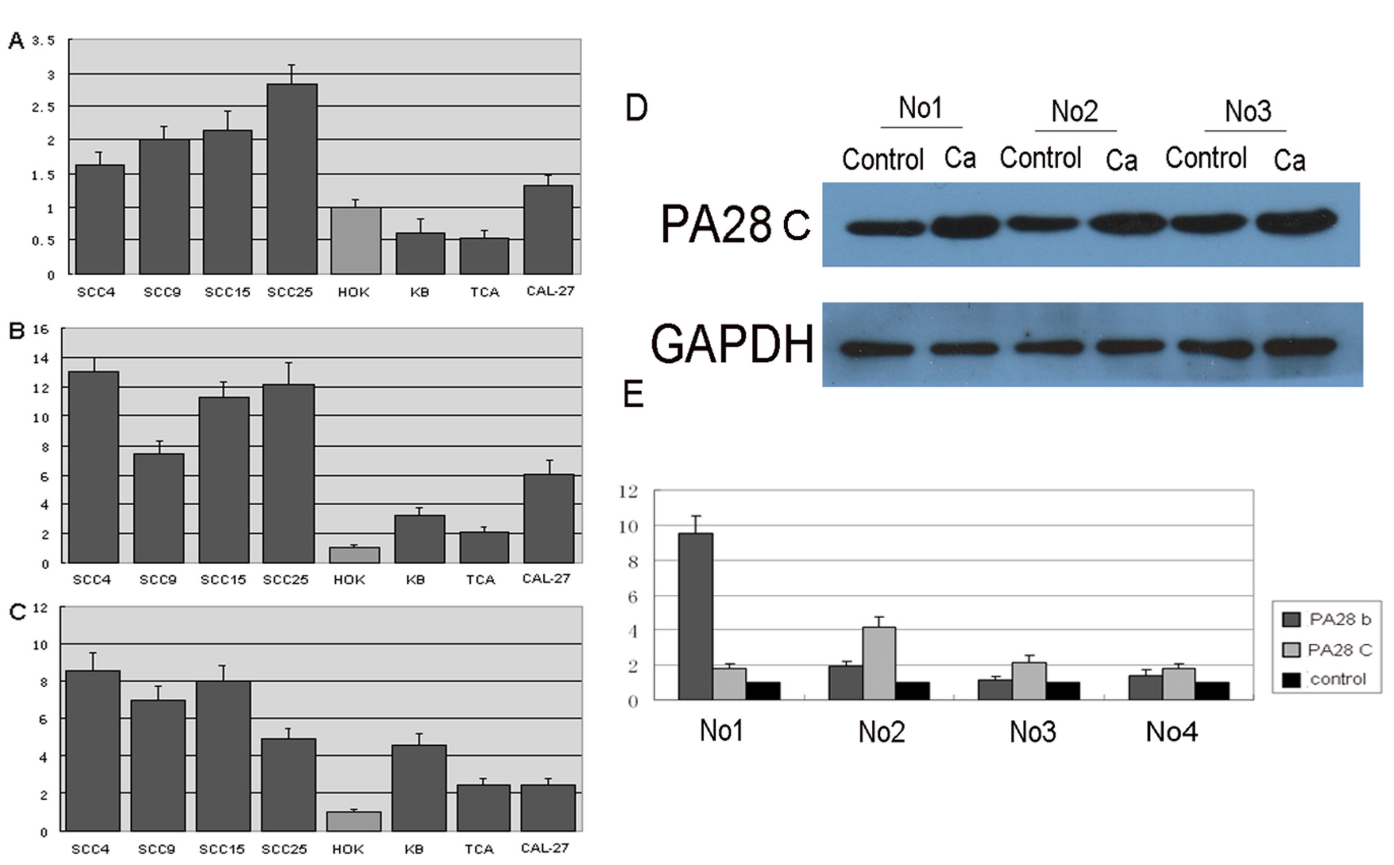
**Validation of PA28 in OSCC tissues and cells relative to control oral leukoplakia tissue and normal keratinocytes**. (A, B, C) The mRNA expression evaluated by real-time PCR in seven OSCC cell lines when compared to the oral keratinocytes. The mRNA level of the three PA28 homologs, a, b and g were shown in A, B, and C, respectively. Except PA28 a in KB and Tca8113 cell lines, the mRNA expression in OSCC cells were reproducibly increased. (D) Crops of images of quantitative measurement of PA 28 g protein in 3 pairs of OSCC tissues (Ca) and oral leukoplakia tissues (control) by Western blotting analysis using PA 28 g-specific antibody. (E) The mRNA expression evaluated by real-time PCR in 4 pairs of OSCC tissues and oral leukoplakia tissues.

Evaluation of PA28 expression in seven OSCC cell lines relative to the human immortalized oral keratinocytes (HOK16E6E7) and of PA28 expression in OSCC tumor tissues versus oral leukoplakia tissues was performed by real time RT-PCR and Western blotting. Consistent with observations from 2-DE, expression of PA28 was markedly increased at both the mRNA and protein levels in OSCC cells and tumor tissues compared with normal keratinocytes and oral leukoplakia tissues (Figure 7).

## Discussion

Only a few studies were done on proteomic analysis of the malignant transformation mechanism from precancerous lesion with different stage of dysplasia into invasive cancer. We have performed a comparative proteomic analysis to profile differentially expressed proteins in the transformation process. Using GO analysis, we further analyzed the biological process and pathway network of these proteins, which can generate a new insight into systemic biology in carcinogenesis.

Oral squamous carcinoma, like esophageal adenocarcinoma, has been associated with the presence of precancerous lesion with different stage of dysplasia, thus providing a good model to elucidate every stage of carcinogenesis in more detail. In our study, each pair of precancerous and cancer tissues was from the same patient, which provides an opportunity to eliminate or at least reduce heterogeneity. Using the proteomic approach, we have identified 85 differently expressed gene products and found some proteins are related to apoptosis, response to stimulus, metabolic regulation and etc. We can thus conclude that these proteins may play an important role in malignant transformation process. Our most significant finding was that several proteins in the same protein families and homologs were identified in this transformation process, such as peroxiredoxins (Peroxiredoxin-3 and 4), Annexin family (A1, A 3, A 4, A 5, A 8), Rho GDP-dissociation inhibitor 1 and 2, Heat shock protein family (70 kDa protein 1, 71 kDa protein, and Heat shock protein beta-1), PA28 homolog (PA28 a and b), Protein S100 family (A7, A8, A9, A10, A11, and A16). Among which, the annexins and S100 are two super-families of closely related calcium and membrane-binding proteins and their relationship with carcinogenesis has been widely studied. They have a diverse range of cellular functions including vesicle trafficking, cell division, apoptosis, calcium signaling and growth regulation.

Many studies have revealed the annexins to be among the genes whose expression is differentially altered in neoplasia. Some annexins showed increased expression in specific types of tumors, while others displayed loss of expression. In our report, the expression level of annexin A1, 3, 4, 5, 8 were all decreased while annexin A8 showed increased expression. Annexin A1 has been extensively studied *in vitro *and *in vivo*. The loss of expression of annexin A1 in our study confirmed previous findings in head and neck squamous carcinomas[12]. Expression of annexin A3 has only been studied in a limited number of tumor types with only one report regarding its expression in head and neck cancer [13]. For annexin A4, a few studies reported its increased expression in clear cell renal cancer and colorectal cancer by using a combination of proteomics tools [14,15]. The change of Annexin A5 was also observed in our study, which has been considered as one of the signals on the surface of the apoptotic cells and has been used as a probe for apoptosis [16]. Annexin A8 has been shown to be consistently over-expressed in acute promyelocytic leukaemia, breast cancers, pancreatic cancer by a combination of gene expression microarrays and immunohistochemistry. The expression of annexin A4, 5, and 8 in head and neck cancer has been reported in this study for the first time, which was consistent with the results obtained in other studies concerning their expression in other cancers [17,18].

The S100 proteins are a multi-gene calcium-binding family of proteins comprising 20 known human members. There has been growing interest in the S100 protein family and their relationship with different cancers. While the precise role of S100 proteins in the development and promotion of cancer remains unclear, it is evident that the S100 proteins have a variety of intracellular and extracellular roles, and that disruption of any one of these functions may contribute to carcinogenesis. There is evidence that these proteins play a major role in tumor metastasis by interacting with a number of different proteins, including matrix metalloproteinase, cytoskeletal proteins, p53, Jab1, Cox-2 and BRCA1. In this study, we have identified a series of members including S100A7, 8, 9, 10, 11, and 16 with differential expression in OSCC tissues. S100A7 (psoriasin) was a member first characterized as being highly expressed in psoriatic keratinocytes [19]. There is accumulating evidence that S100A7 is up-regulated in bladder cancer skin tumors and some invasive carcinomas. Its expression is associated with a poorer prognosis and reduced survival [20-22]. On the contrary, other reports in OSCC showed its expression was associated with a better prognosis based on the finding that S100A7 is highly expressed in pre-invasive, well-differentiated and early staged OSCC, but little or no expression was found in poorly differentiated, later-staged invasive tumors [23]. Other reports showed that S100A7 inhibits both OSCC cell proliferation in vitro and tumor growth/invasion in vivo [24]. These results were echoed by our study, in which S100A7 was identified to be down-regulated in the transformation process form precancerous dysplasia to invasive cancer. Therefore, unlike in other tumors, our data suggests S100A7 to be a tumor suppressor in OSCC. The detailed function should be further elucidated. S100A8 and S100A9 which form a heterodimer complex 90 are up-regulated in many cancers and have been implicated in the metastatic process including gastric cancer, prostate cancer, colorectal cancer, and breast cancer [25-27]. In OSCC, one study reported there was more than a 10-fold over-expression of S100 A8 in HPV18+ OSCC [28]. For S100A11, its function has been somewhat controversial. In bladder carcinoma and renal carcinoma, its expression is related to tumor suppression, and decreased expression of S100A11 has been associated with an increase in histopathological grade, poorer prognosis and decreased survival [29]. However, in prostate cancer and breast cancer it is thought to be a tumor promoter. Its increased expression in prostate cancers has been shown to be associated with advanced pathological stage [30]. There is only one report about the gene expression of S100A11 related to its diverse functions [31]. S100A16 protein, a new and unique member of the EF-hand Ca (2+)-binding proteinswas found to accumulate within nucleoli and be translocated to the cytoplasm in response to Ca (2+) stimulation [32]. Here we report for the first time the expression of S100A16 protein in carcinogenesis from precancerous dysplasia to OSCC. It is possible that each S100 protein may play multiple roles in tumourigenesis and metastasis. This highlights the need for an improved understanding of the S100 family, before the design of S100 protein-targeted therapies can be achieved.

Proteasomes are large complexes that carry out crucial roles in many cellular pathways by degrading proteins in the cytosol and nucleus of eukaryotic cells [33]. Proteasomes are activated by protein complexes that bind to the end rings of subunits. PA28 (also known as 11S or REG) has been shown to bind specifically to and activate 20S proteasomes against model peptide substrates [34]. The biological roles of PA28 are less well understood. There are three PA28 homologs, called a, b and g. Although PA28a and b subunits are expressed in many organs, they are particularly abundant in immune tissues and are virtually absent from the brain. By the late 1990s, PA28ab was found to contribute to Class I presentation, based on the high levels of PA28ab in immune tissues, the IFNg induction of PA28ab and many components of the class I pathway, and the direct production of some Class I epitopes by PA28ab-proteasome complexes[35,36]. Our results also reinforce the connection between PA28ab and cellular immunity by showing that the key proteins in the MHC I antigen presenting pathway like PA28, proteosome, HSP70 and HSP90 have been detected up-regulated in OSCC tissues. An earlier study showed that PA28g expression correlated with cell proliferation. Recently, some researchers have gained more insight into the role of PA28g in apoptosis [37]. These findings were paralleled by studies suggesting that PA28g functions in cell cycle progression and has an immune role [38]. Two-hybrid screens have identified several proteins that interact with PA28g as well. Interestingly, all these findings suggest that PA28g is an anti-apoptotic factor. Less is known about how PA28g may suppress apoptosis in oral carcinesogenesis. In our validation study, Three homologs were all included. The results have confirmed the up-regulation of PA28 in carcinogenesis by comparison between several OSCC cell lines and oral keratinocytes. In our further studies, we would valuate the PA28 ab complex and PA28 g immunostaining pattern in different stage of tissue samples from normal, precancerous to infiltrative OSCC. Moreover, the relationships of immunostaning with survival rate and recurrence will be analyzed.

## Conclusion

In summary, we have applied proteomic technologies to analyze the malignant transformation from precancerous oral leukoplakia to oral cancer from 6 patients, and we have identified 85 different proteins with altered expression levels in OSCC in the transformation, of which 53 were up-regulated. Previous characterizations regarding their functions and possible interactions with other proteins and in particular the pathways involved were also evaluated. As a key factor in tumor metastasis, PA was chosen for transformation at first. The proteosome activator PA28 was studied for their expression and interactive networks correlated with oral malignancy. We have being started the further research on other potential biomakers like peroxiredoxins, annexin family and S100 family. This is an example of a systems biology study, in which functional proteomics was constructed to help to elucidate mechanical aspects and potential involvement of proteins of interest in biological pathways.

## Methods

### Cell Culture

Seven oral squamous cell carcinoma cell lines (SSC4, SCC9, SCC15, SCC25, Cal 27, KB, Tca8113) were maintained in Dulbecco's modified Eagle's medium (Invitrogen) containing 10% fetal calf serum (Invitrogen), 100 units/liter penicillin, and 10 mg/liter streptomycin. HOK16E6E7 cells, a human immortalized oral keratinocyte cell line, was cultured in keratinocyte growth medium containing 0.15 Mm calcium and supplemented with epidermal growth factor (Invitrogen). All cell lines were maintained at 37°C in an atmosphere containing 5% CO2.

### Tissue collection and sample preparation

Six pairs of tumors and oral leukoplakia tissues with dysplasia were obtained from six patients in West China Stomatological Hospital, Sichuan University. The specimens were examined histologically by hematoxylin and eosin (HE) staining, and the clinicopathologic stage was determined according to the TNM classification system of the International Union against Cancer [39]. Patients receiving previous chemotherapy or radiation treatment were excluded. All the tissue specimens were snap-frozen in liquid nitrogen for proteomic analysis. Hematoxylin-stained 5 μm frozen sections were reviewed by a Board-Certified pathologist (Y. Chen) for tumor cellularity (oral squamous carcinoma) or oral leukoplakia mucosa (moderate-high grade dysplasia). Informed consent was obtained from all patients or their relatives for the experimental use of their tissues. Medical records were reviewed and data were coded to protect patient confidentiality. The project was approved by the Scientific and Ethics Committee of Sichuan University.

### Two-Dimensional Electrophoresis

Two-dimensional gel electrophoresis was performed essentially as previously described [40]. The protein concentration of the supernatants was determined using a Bio-Rad protein kit. All the paired samples were quantitatively analyzed in group. Samples of 1 mg protein were applied on immobilized pH 3–10 nonlinear gradient strips in sample cups at their basic and acidic ends. Focusing started at 200 V and the voltage was gradually increased to 8000 at 4 V/min and kept constant for a further 3 h (approximately 150 000 Vh totally). The second dimensional separation was performed in 12% SDS-polyacrylamide gels. The gels (180 × 200 × 1.5 mm^3^) were run at 40 mA/gel. After protein fixation in 50% methanol containing 5% phosphoric acid for 2 h, the gels were stained with Coomassie Brilliant Blue R-250 (Merck, Germany) for 12 h and the protein spots were visualized. Each experiment was performed twice to ensure the accuracy of analyses. The images were scanned using a Bio-Rad high quality white light GS-800 scanner (400–750 nm). The differentially expressed proteins were identified using the PD-Quest 2DE analysis software (Bio-Rad, USA). The quantity of each spot in a gel was normalized as a percentage of the total quantity of all spots in that gel and evaluated in terms of O.D. The student's t-test was applied to compare the spot relative volume between two groups. Significant spots that showed changed consistently and at least 2.0-fold difference (p < 0.05) were selected for tandem mass spectrometry (MS/MS) analysis.

### Protein identification by nano-HPLC-ESI-Q-TOF-MS/MS

The protein spots were excised manually and digested using sequence grade trypsin (V511A, Promega). The protein samples were reduced, alkylated, and then digested with trypsin using standard protocols as previously described. The digests were analyzed using a nano-HPLC system coupled to Q-TOF Primer mass spectrometer (Q-TOF, Micromass, Micromass, Manchester, UK) equipped with an electrospray ionization source. Spectra were accumulated until a satisfactory signal/noise ratio had been obtained. Only double, or more than double, charge peaks, in the mass range from 400 to 1600 m/z, were considered for MS/MS. Ions exhibiting a detection intensity exceeding 10 counts/second were selected for production of ion spectra by Collision Induced Dissociation (CID). Trypsin autolysis products and keratin-derived precursor ions were automatically excluded. Three MS/MS ions were selected for each survey scan. All data used to extract peak information, which was used to create the MS/MS peak list, were generated from one combined spectrum. The tandem mass spectrometry (MS/MS) data, "pkl list (pkl) " files acquired by the software of ProteinLynx 2.2.5 (Waters), included the mass values, the intensity and the charge of the precursor ions (parent ions with +2 or +3 charge in this study). The pkl files were analyzed using MASCOT search engine  against SWISS-PROT protein database. Proteins were identified on the basis of two or more peptides whose ion scores both exceeded the threshold, *P *< 0.05, which indicated the 95% confidence level for these matched peptides [41].

### Functional genome (Gene) ontology analysis

To translate sets of differentially regulated genes into functional profiles, we applied GOTree Machine  for data analysis. GOTree Machine generates a GOTree, a tree-like structure to navigate the Gene Ontology-directed acyclic graph for input protein sets, and to provide navigational data and visualization. The software categorizes gene products based on the location of the protein within cellular components and suggests possible biochemical, biological, and molecular functions. Statistical analysis was performed to identify the most important GO categories for the input protein sets and to suggest their potential biological importance in each of the categories. The tissue expression pattern diagram was constructed with the software too.

### Biological association network

Changes in gene/protein expression are not isolated events. Therefore, we hypothesize that differentially regulated genes/proteins that have interacted with others may be more likely to play crucial roles in oncogenesis. To identify those key factors, we explored the biological associations among the differentially expressed proteins using String . Differentially expressed proteins each identified with a SWISS-PROT number were uploaded into String. The proteins were integrated into biological association networks based on protein interactions documented in a curated database

### Real-Time Quantitative PCR

Real-time PCR was performed on a LightCycler (Roche Diagnostics) platform as previously described [42]. The following forward and reverse primers were selected by using PRIMER EXPRESS software and were synthesized by TaKaRa Co. Ltd. PA28 a F: 5'-GCC AAC TTG AGC AAT CTG A-3'; PA28 a R:5'-ACA GGG AGG ACC TTT GTC-3'; PA28 a TM: 5'-FAM-AGAAAGAGGAGCGGAAGAAACAGC-ECLIPSE-3'; PA28 b F: 5'-TAG CGA CTG AAG CAG CAT G-3'; PA28 b: 5'-GGA AGT CAA GTC AGC CAC AT-3'; PA28 b TM: 5'-FAM-ATT CCT CTA CAG ATT CTT GCC ACA GA-ECLIPSE-3'; PA28 g F: 5'-AGA AGA CTT GGT GGC AAA T-3'; PA28 g R: 5'-TCC AGT CCA TCA TGG CTA T-3'; PA28 g TM: 5'-FAM-CAT GAA TCT CCC AGT CCC TGA CCC-ECLIPSE-3'. A SYBR green PCR kit (Applied Biosystems) was used by following the manufacturer's instructions, and the analyses were performed in duplicate or triplicate. Target mRNA values were normalized by using β-actin mRNA as an internal control.

### Western Blotting

Protein extracts were prepared using a lysis buffer containing protease inhibitor mixture 8340. For Western blotting analysis, 20 ug of protein were separated by 12% SDS-PAGE, then transferred to a PVDF membrane (Millipore), and probed with monoclonal mouse anti-PA28 g antibody (1:1000; Cell signaling Technology). The blots were labeled with horseradish peroxidase-conjugated secondary antibodies (1:10,000) and visualized with an ECL detection system (Pierce).

### Statistical Analysis

All samples were processed twice by 2-D gels and real-time PCR. The results of the individual analyses correlated well with each other without unexpected deviations. The comparison was done by unpaired Student's t test. Significance was set a p < 0.05. Computations were performed using the SPSS version 11.5 software package.

## Authors' contributions

*ZW and XDF *contribute equally to conducting the experiments and writing the paper, *XYL *contributed to the genome (Gene) ontology analysis and network analysis, *LJ and XZ *contributed to expression validation, *NJ and JL *contributed to Two-Dimensional Electrophoresis and trypsin digestion, *QMC *and *LJL *provided the original concept of the study, supervised the study and contributed to writing the paper. All authors read and approved the final manuscript.

## Supplementary Material

Additional file 1 **The enlarged and cropped images of 11 selected protein spots in 2D Gel**. 11 proteins were selected as examples showing the consistent expression changes in enlarged form. The images of each changed protein spot were compared with the control. (A) 2-DE gel images of 11 selected protein indicated by arrows in the panels. Each panel shows an enlarged view of the gel spots from Figure 2. (B) Volume density analysis graphs: the data were expressed as mean ± SD of twelve repeats. Click here for file
