# Supplementary figures and images for: Involvement of potential pathways in malignant transformation from Oral Leukoplakia to Oral Squamous Cell Carcinoma revealed by proteomic analysis
Source: BMC Genomics. 2009 Aug 19;10:383. doi: 10.1186/1471-2164-10-383 (PMC2746235; doi:10.1186/1471-2164-10-383)

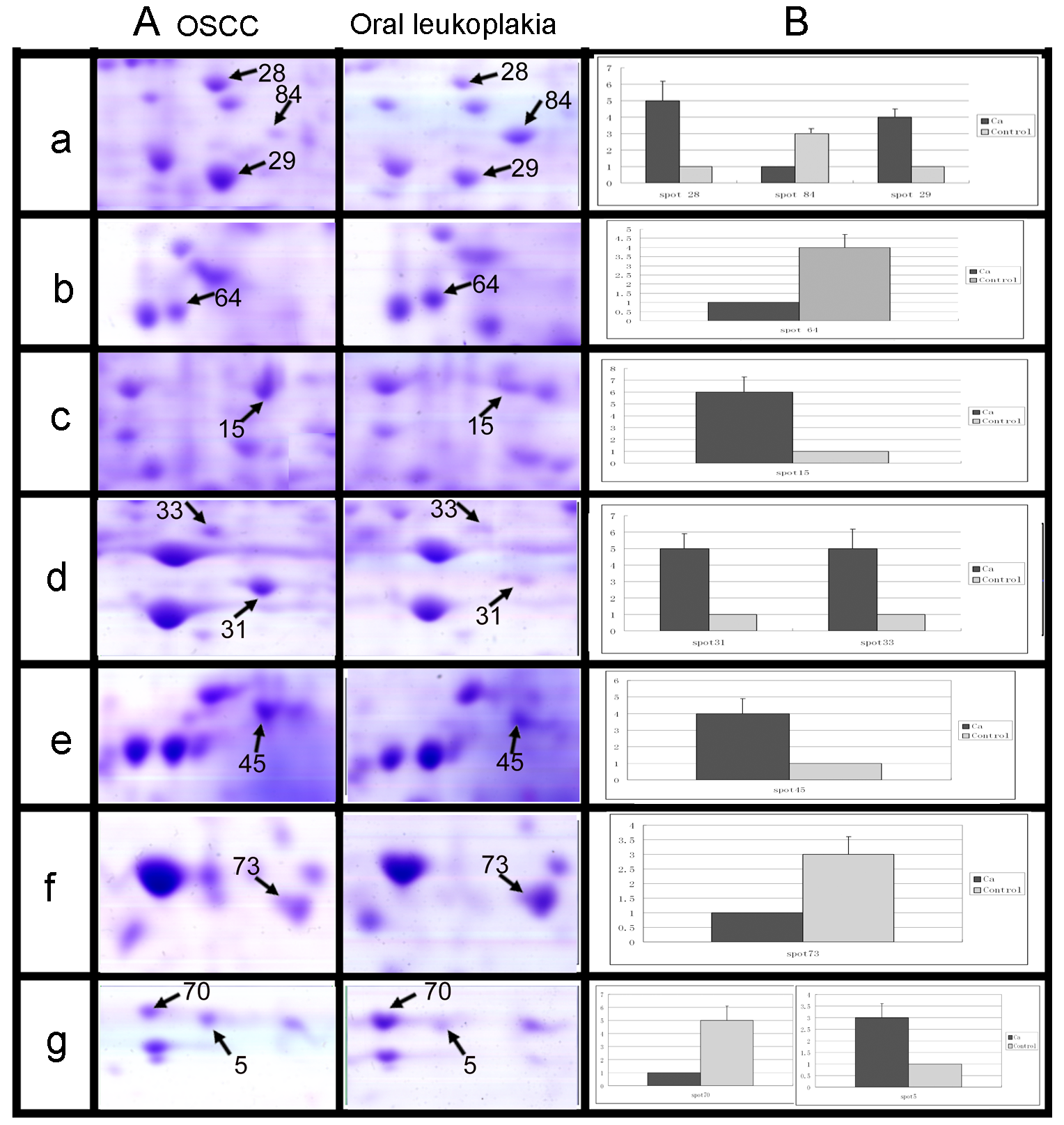

Supplement: Additional file 1 — The enlarged and cropped images of 11 selected protein spots in 2D Gel. 11 proteins were selected as examples showing the consistent expression changes in enlarged form. The images of each changed protein spot were compared with the control. (A) 2-DE gel images of 11 selected protein indicated by arrows in the panels. Each panel shows an enlarged view of the gel spots from Figure 2. (B) Volume density analysis graphs: the data were expressed as mean ± SD of twelve repeats. [file 1471-2164-10-383-S1.tiff]
